# Supplementary material for: “The child of your fellow is your child”: Building on existing protective norms to engage men as caregivers; qualitative findings from an exploratory evaluation of an edutainment intervention to prevent age-disparate transactional sex
Source: PLoS One. 2025 May 2;20(5):e0321191. doi: 10.1371/journal.pone.0321191 (PMC12048162; doi:10.1371/journal.pone.0321191)
Supplement: S1 Checklist — (DOCX) [file pone.0321191.s001.docx]

Inclusivity in global research

PLOS’ policy on inclusivity in global research aims to improve transparency in the reporting of research performed outside of researchers’ own country or community and ensures that PLOS publications reporting global research adhere to high standards for research ethics and authorship. Authors of relevant research articles may be asked to complete the questionnaire below, which outlines ethical, cultural, and scientific considerations specific to inclusivity in global research. This questionnaire may be requested when researchers have travelled to a different country to conduct research, if research uses samples collected in another country, research with Indigenous populations or their lands, or if research is on cultural artefacts. Researchers travelling to another country solely to use laboratory equipment will not normally be required to complete the questionnaire. However, the questionnaire can be requested at the journal’s discretion for any submission – if you have been requested to complete this questionnaire by the PLOS journal you submitted to, please do so.

Please complete the questionnaire below and include this as a Supporting Information file with your manuscript. Note that if your paper is accepted for publication, this checklist will be published with your article in the supporting information files. Please ensure that you reference the checklist in the main body of your manuscript. We suggest adding a subsection ‘Inclusivity in global research’ to your Methods section and adding the following sentence: “Additional information regarding the ethical, cultural, and scientific considerations specific to inclusivity in global research is included in the Supporting Information (SX Checklist)”

The questions have been designed to be applicable to a wide range of study types, and there are subsections for both human subjects research and non-human subjects research. If any of the questions are not relevant to your research please mark them as “N/A” as appropriate.

**Ethical considerations, permits and authorship**

*This section is applicable to all research types.*

Provide details as to who granted permissions and/or consent for the study to take place in the Methods section of your manuscript. This should include the names of **all** ethics boards, governmental organizations, community leaders or other bodies that provided approval for the study. If individuals provided approval refer to these people by their role or title but do not list their name(s).

Reported on page number: 15

If there were any deviations from the study protocol after approval was obtained please provide details of these changes in the Methods section of your manuscript.
Did this study involve local collaborators that are residents of the country where the research was conducted or members of the community studied? If you do not have any authors from said communities, please provide an explanation for this below.

Reported on page number: N/A

Yes, our local collaborators were Amani Girls Organization (AGO), who led on research, and the Tanganyika Christian Refugee Service (TCRS), who led on implementation. Both are local NGOs fully-staffed with Tanzanians. Three staff members from these organizations met the PLOS criteria for authorship: Veronicah Gimunta and Revocatus Sono from AGO, and Oscar Rutenge from TCRS.

Everyone listed as an author should meet PLOS’ criteria for authorship and all individuals who meet these criteria should be included in the author byline, rather than the acknowledgements. For further information please see the journal’s Authorship Policy.

**Human subjects research (e.g. health research, medical research, cross-cultural psychology)**

Did you obtain written informed consent from a representative of the local community or region before the research took place? How did you establish who speaks for the community? Details of written informed consent obtained from study participants should be reported separately in the Methods section of your manuscript.

We obtained a research ethics approval certificate to conduct this study from the National Institute of Medical Research (NIMR), which is a parastatal organization under the Ministry of Health, Community Development, Gender, Elderly and Children. The population included in the study were the 331 households that had received solar-powered radios from TCRS through previous programming, and thus, were familiar with the organization. Having worked with this population extensively, TCRS also has a close working relationship with the district council departments headed by the District Executive Director under the Ministry of Regional Administration and Local Government. Before the start of our study TCRS shared official letters, which included the local ethics approval from NIMR as well as a copy of the radio drama, with the Kishapu District Executive Director to inform them of the study.

How did members of the local community provide input on the aims of the research investigation, its methodology, and its anticipated outcome(s)?

Potential participants were given an information sheet in Kiswahili, which was then read with the researcher to enable them to ask any questions before consenting to participate in the study. A verbal comprehension questionnaire was then provided to ensure that the information was understood correctly. If the individual was unable to read or write, an impartial witness was also present during the consent process to help them understand the documents.

When engaging with the local community, how did you ensure that the informed consent documents and other materials could be understood by local stakeholders?

The LINEA radio drama was co-created by the London School of Hygiene & Tropical Medicine (LSHTM), AGO and Media for Development International in Tanzania. AGO, a Tanzanian-based NGO, also led all research for this study, and therefore co-designed the aims, methods and anticipated outcomes, with support from LSHTM. They also worked closely with TCRS to ensure that the research questions and methodologies were appropriate for the specific population that we worked with in Shinyanga region, Kishapu district.

Will the findings of the research be made available in an understandable format to stakeholders in the community where the study was conducted (e.g. via a presentation, summary report, copies of publications, etc.)? Please provide details of how this will be achieved.

Yes, we plan to organize a dissemination meeting in the community during which we will share the results of our research via a presentation.

**Non-human subjects research using specimens/ animals collected as part of the study, or those housed in archival collections. Examples include archaeology, paleontology, botany and zoology.**

Did the permission you obtained from a local authority to perform the study include an agreement on access to outputs and benefit sharing? This may include procedures to enable fair distribution of the benefits and resources arising from the research performed. Please include any details of Prior Informed Consent and Benefit Sharing Agreements obtained. These may be required by field-specific regulations, for example the Convention on Biological Diversity (CBD) and the associated Nagoya Protocol.

N/A

If the material used in your study was imported, please A) provide the year it was imported and B) indicate whether permits were obtained to import/export the materials used, C) provide details of any permits obtained. If this information is not available, please indicate this.

N/A

If you used archival specimens, please state how the material used in your study was acquired by the institute it is held in and provide details of any permits obtained for the original excavations/ sample collection. If this information is not available, please indicate this.

N/A

How was the potential cultural significance of the materials collected in your study to local communities considered in your research design? Were Indigenous peoples and/or local researchers and institutions involved with archaeological excavations / collection of specimens? If so, please provide a description of their involvement.

N/A

If your manuscript includes photographs of human remains please indicate whether authors obtained permission from descendants or affiliated cultural communities to do so.

N/A
