# Supplementary material for: “The child of your fellow is your child”: Building on existing protective norms to engage men as caregivers; qualitative findings from an exploratory evaluation of an edutainment intervention to prevent age-disparate transactional sex
Source: PLoS One. 2025 May 2;20(5):e0321191. doi: 10.1371/journal.pone.0321191 (PMC12048162; doi:10.1371/journal.pone.0321191)
Supplement: S1 File — (PDF) [file pone.0321191.s002.pdf]

LINEA
BASELINE - RADIO DRAMA: <u>ADULT MEN AND WOMEN</u>
INDIVIDUAL INTERVIEW TOPIC GUIDE THEMES AND EXAMPLE QUESTIONS

<u>KISHAPU</u>

**GENERAL QUESTIONS**
Example question:<br><br>Is there anybody you admire in your community? Who are they?<br><br>*If they cannot think of anyone probe with examples, such as: family member, friend, colleague, local business person / politician…*<br><br>*For one-word answers, probe with:*<br><br>i. What do you admire about this person? Why?
**GENDER**
Example question:<br><br>What do you think are the main challenges girls face in this community?<br><br>*Let them express themselves freely, then probe if necessary:*<br><br>a. Can you give me examples?<br><br>b. How do adolescent girls respond to these challenges?
**TRANSACTIONAL SEX – BELIEFS AND ATTITUDES**
Example question:<br><br>Can you tell me about what happens in transactional sex relationships between adult men and girls aged 13-15?<br><br>*Let the participant express themselves freely. Then ask:*<br><br>a. How do you think these relationships start?<br><br>*Probe: Explore gift giving: is it men's initiative, do girls ever provide a signal to men?*<br><br>b. Can you give me examples?

TRANSACTIONAL SEX EXPERIENCES – MEN
Example question:<br><br>We have been speaking about transactional sex in your community. Has anything like this ever happened to you?<br><br>*Let them express themselves freely, then probe if necessary.*<br><br>a. Have you ever offered a 13–15-year-old girl who is not in your family gifts or favours?<br><br>*If "**yes**", ask:*<br><br>i. Can you tell me about this experience? What happened?<br><br>*Let them express themselves freely, then probe if necessary: how did they meet? how did the relationship develop? Is it still ongoing? If not, how long did it last? How did it end?*<br><br>ii. How old was/is the girl?<br><br>iii. How do you think about this experience now?<br><br>*Let them express themselves freely, then probe: happy, regret…?*
**TRANSACTIONAL SEX – SOCIAL NORMS**
Example question:<br><br>What do people in your community think about men who take part in transactional sex with girls (aged 13-15)?<br><br>*Let them express themselves freely, then probe around community judgements if necessary. For example, do people admire these men? Do they think that these men care about girls?*
**CLOSE THE INTERVIEW**
Example question:<br><br>What do you think could be done to support girls to avoid transactional sex?<br><br>*Let them express themselves freely, then if necessary probe around support at school, at home, by the government...?*<br><br>a. Who in the community can support them, and how?

Let them express themselves freely, then probe around the answers that they didn't ask about:

i. How can ***men*** support girls to keep them from engaging in transactional sex?

ii. How can ***women*** support girls to keep them from engaging in transactional sex?

iii. How can ***teachers*** support girls to keep them from engaging in transactional sex?

iv. How can girl's ***peers*** support them and keep them from engaging in transactional sex?
