# Supplementary material for: “The child of your fellow is your child”: Building on existing protective norms to engage men as caregivers; qualitative findings from an exploratory evaluation of an edutainment intervention to prevent age-disparate transactional sex
Source: PLoS One. 2025 May 2;20(5):e0321191. doi: 10.1371/journal.pone.0321191 (PMC12048162; doi:10.1371/journal.pone.0321191)
Supplement: S2 File — (PDF) [file pone.0321191.s003.pdf]

LINEA
ENDLINE - RADIO DRAMA: ADULT MEN AND WOMEN
INDIVIDUAL INTERVIEW TOPIC GUIDE THEMES AND EXAMPLE QUESTIONS

KISHAPU

**RADIO DRAMA**
Example question:<br><br>Which radio drama characters did you like most?<br><br>*Let the participant express themselves freely and then ask:* Why?
**HOUSEHOLD DISCUSSION SESSIONS**
Example question:<br><br>How was it to discuss the radio drama with your daughter/other members of the family?<br><br>*Let the participant express themselves freely, and then ask:*<br><br>a. Did you feel comfortable discussing it with them? Why yes/Why not?<br><br>b. Did you feel able to share your views with / in front of your family members? Why yes/Why not?<br><br>c. Were there any problems with discussing the radio drama with your family?
**TRANSACTIONAL SEX – BELIEFS & ATTITUDES**
Example question:<br><br>Can you tell me how transactional sex relationships between adult men and girls aged 13-15 start?<br><br>*Probe: Explore gift giving: is it men's initiative, do girls ever provide a signal to men?*<br><br>a. What happens in these relationships?<br><br>*Let the participant express themselves freely. Then ask:*<br><br>b. Can you give me examples?

**TRANSACTIONAL SEX EXPERIENCES – MEN**
Example question:<br><br>We have been speaking about transactional sex in your community. Has anything like this ever happened to you?<br><br>*Let them express themselves freely, then probe if necessary.*<br><br>a. Have you ever offered a 13–15-year-old girl who is not in your family gifts or favours?<br><br>*If "**yes**", ask:*<br><br>i. Can you tell me about this experience? What happened?<br><br>*Let them express themselves freely, then probe if necessary: how did they meet? how did the relationship develop? Is it still ongoing?*<br>*If not, how long did it last? How did it end?*<br><br>ii. How old was/is the girl?<br><br>iii. How do you think about this experience now?<br><br>*Let them express themselves freely, then probe: happy, regret…?*
**TRANSACTIONAL SEX – SOCIAL NORMS**
Example question:<br><br>What do people in your community think about men who take part in transactional sex with girls (aged 13-15)?<br><br>*Let them express themselves freely, then probe around community judgements if necessary. For example, do people admire these men? Do they think that these men care about girls?*
**CLOSE THE INTERVIEW**
Example question:<br><br>Did any of the radio drama storylines or characters inspire you to change anything in your life?<br><br>*Ask about their role in the community, work, family life.*
