# Supplementary material for: “The child of your fellow is your child”: Building on existing protective norms to engage men as caregivers; qualitative findings from an exploratory evaluation of an edutainment intervention to prevent age-disparate transactional sex
Source: PLoS One. 2025 May 2;20(5):e0321191. doi: 10.1371/journal.pone.0321191 (PMC12048162; doi:10.1371/journal.pone.0321191)
Supplement: Attachment — Submitted filename: Response to Editors Comments_12 September 24.docx [file pone.0321191.s004.docx]

12 September, 2024

PLOS ONE – Response to Editor Comments

PONE-D-24-32227
“The child of your fellow is your child”: Building on existing protective norms to engage men as caregivers; qualitative findings from an exploratory evaluation of an edutainment intervention to prevent age-disparate transactional sex

| **Editor Comments** | **Author Responses** |
| --- | --- |
| 1. Your paper presents findings from in-depth interviews with a sample of 18 adult men who participated in a radio-drama intervention designed to reduce of the risks of transactional sex with adolescent girls in Tanzania. Before sending this out for review, I have some suggestions for revision that will make the review process go smoother.  My reading suggests that the study gave 331 low-income households the opportunity to listen to the drama on a weekly basis and met with the households to discuss the drama. For this study, 81 members of those households were invited to be interviewed at both the beginning and end of the intervention, including the 18 men who were the focus of this paper.  You provide examples of how the men felt about the role of men as potential change agents in the community to prevent transactional sex among adolescent girls with a focus on the post interviews. However, I have several suggestions for ways to make the results easier to follow and to interpret. | Thank you very much for reviewing our paper. We look forward to addressing your suggestions. |
| 2. First, it is not clear whether the intervention was merely listening to the drama or of having discussions led by the research team on a weekly basis. Those discussions should be described if they were part of what the 18 men were exposed to. If those discussions focused on the themes of interest, then it is not the drama per se, but the drama as a way to provoke conversations with the researchers that is being studied. It would be preferable if those weekly discussions did not occur for the 18 men, so that one can draw conclusions about the drama as an intervention in itself.   Otherwise, those discussions should be described as part of the intervention. | Thank you for raising these important points. All 331 participating households were exposed to the radio drama, while a sub-set of 60 households were randomly sampled to take part in the household discussion sessions. The aim of this in the overall study was to explore whether these discussions had an impact above and beyond that of the radio drama alone. The overall impact evaluation paper found no clear evidence that the discussion sessions had an additional impact. This has been clarified on p 13. Likewise in this paper we were not able to detect any differences between those men who received the household discussion sessions and those who did. This has also been added to the results section on p 13.  The discussion sessions were conducted with the families of 13 out of the 18 men included in the current study. This has been added on p 14. We have also added whether each man quoted was assigned to receive household discussion sessions or not in the label at the end of each quote throughout the results.  In the results section about the radio drama catalysing discussions about age-disparate transactional sex, while the participants report initiating these discussions on their own while listening to the radio drama, it is also possible that the facilitation of these conversations was role modelled to them during the household discussion sessions, although we found evidence of this even in men not assigned to receive the household discussion sessions. These reflections have been added on p 23-24 of the results and p 32 of the discussion.  Following your suggestion, we have added additional description of the household discussion sessions on p 11.  In the discussion section on p 31 and conclusion section on p 35 we have also added mention of the household discussion sessions explicitly as part of the intervention. |
| 3. Another aspect of the study is the intervention itself. Can you provide some examples of how the drama featured the themes of interest? What story lines illustrated the need for men to take more responsibility for preventing transactional sex? | Thank you for this great suggestion. Various story lines within the radio drama illustrated the need for men to take more responsibility for preventing transactional sex in different capacities.  From one of the teachers at school who realizes that a colleague is engaging in age-disparate transactional sex with adolescent students and intervenes, to a fellow bodaboda driver who advises one of the main characters to stay away from age-disparate transactional sex given his own experience, as well as one of main characters, Tuma, who ultimately realizes that trying to engage Amali (main girl character) in age-disparate transactional sex is not in Amali’s best interest, and it could ultimately interfere with his own plans and life.  Among these men, the character that speaks directly to male caregivers (and therefore relevant to this paper) is the father of Amali, the central adolescent girl character. At the beginning of the story he abides by traditional, patriarchal norms; often travelling for work and barely communicating with his daughters. After an injury that leaves him unemployed and at home, he becomes more involved in his daughters’ lives, going to speak to their teacher at school (depicted in new figure 1, p12), encouraging their careers, and ultimately helping them avoid age-disparate transactional sex. He also ensures that the teacher who engaged in age-disparate transactional sex with his daughter is accountable and does not harm other girls in the school.  For the sake of brevity we have kept the information to this last point in the paper on pp 10-11, and added a description of the other characters in a new table 1 on p11. |
| 4. It would also be helpful to provide an outline of the interviews. What were the themes that guided the interviews? And what kind of probing was done to clarify the answers to those questions? It would also be helpful to know what the themes were at baseline versus at the end. This would be especially useful for interpreting whether the drama actually changed what the interviewees thought about men’s roles in preventing transactional sex. The results do not make a very clear case that the participants changed their views. | At baseline and endline the IDI topic guides covered the themes of gender, power and age-disparate transactional sex, including participant’s attitudes, beliefs, social norms and experiences. At endline topic guides covered these same themes with the addition of experiences of listening to the radio drama as a family, and of facilitated household discussion sessions.  Throughout the interviews probing was used to glean examples of participant’s personal experiences with the topics covered. This has been clarified on pp 14-15.  We have also included the topic guide themes and example questions as an Appendix. |
| 5. It would help a lot to show examples of changes in particular participants views from baseline to the end. For example, did the men not even think about their roles at baseline but then show evidence of change at the end? Such evidence would be the most convincing that the intervention itself and not more extensive probing at the end was the source of change. | Thank you for raising this point and allowing as to strengthen our paper to convey the robustness of our findings and attribution of change to the LINEA intervention. In answering your comment we would like to highlight three main arguments:   1. While we use a trajectory analysis to describe the change between baseline and endline for a few individuals, our analyses relied primarily on the frequency with which different thematic findings at the aggregate level arose between the two time points and if, at endline participant’s referred to the radio drama characters or storylines as the reason for their attitude or belief, highlighting that the changes were due to the intervention [1]. This has been added on p 17. 2. We provide evidence from baseline and endline to demonstrate change over time in the paper for participant 53, and this has been highlighted on p 26. Additionally, in response to this comment we have added evidence of individual change from baseline to endline for the theme on catalyzing discussions (participant 76 on pp 22-23) and educating men (participant 51 on p 28). 3. Methodologically, interviews were conducted by the same researchers at baseline and endline using the same topic guide (as described in answer to point 4), thus it is unlikely that the changes we saw in participant’s answers were a result of more probing at endline. This has been added on pp 14-15. |

REFERENCE
[1] Grossoehme D, Lipstein, E. Analyzing longitudinal qualitative data: the application of trajectory and recurrent cross-sectional approaches. BMC Res Notes. 2016;9(136). Doi: 10.1186/s13104-016-1954-1. PubMed PMID: 26936266
