# Supplementary material for: “The child of your fellow is your child”: Building on existing protective norms to engage men as caregivers; qualitative findings from an exploratory evaluation of an edutainment intervention to prevent age-disparate transactional sex
Source: PLoS One. 2025 May 2;20(5):e0321191. doi: 10.1371/journal.pone.0321191 (PMC12048162; doi:10.1371/journal.pone.0321191)
Supplement: Attachment — Submitted filename: Minor comments.docx [file pone.0321191.s013.docx]

**PONE-D-24-32227**

**The child of your fellow is your child”: Building on existing protective norms to engage men as caregivers; qualitative findings from an exploratory evaluation of an edutainment intervention to prevent age-disparate transactional sex**

Reviewers comments:

Minor comments

Authors should consider starting a new paragraph with a strong and direct statement. Therefore, the sentence in Line 63 "Others have also questioned..." should be revised. This comment is also relevant for Line 94 and 115.

Concept of Transactional Sex: Authors may consider starting the section "Age-disparate transaction sex" Line 131, with basic concepts of the subject presented in later paragraphs - Line (140 -146) and part of the subsequent paragraph (Line 147 -151) before detailing its consequences (HIV and VAW)

Line 162 - It notes that the evaluation was carried out in two regions of Tanzania - Kigoma and Shinyanga. I have not seen in the paper the rationale to exclude Kigoma results or focusing in Shinyanga only

Contextualization - I lack context specific information of the problem in Tanzania and Shinyanga specifically in regard to age-disparate sex, VAWG and men's involvement as agent for change and care givers. I assume the context may have been described elsewhere as the current paper is indicated to be part of three. However, readers of the current paper will benefit from a brief description of the same

Methodology

The radio drama "Msichana wa Kati" that was designed to be played in full for nine months (Line 194 - 196) - when did it start? Did it run for nine months as required? On the other hand, authors indicate that baseline evaluation (conducted before the intervention) and endline were 2 -3 months apart (Line 183 -184). There is a date mention in Line 254 that seem to inform the intervention did run long before the baseline assessment - which seem confusing. There is a need for authors to provide clear description on this to warrant intervention replicability.

Discussions

Structured household discussions and its impact - While the authors have indicated (in response to earlier review comment comment 2) that discussions had no added impact, the team presents conflicting conclusions in line 655 - 659 and recommendation Line 659 - 661.

Limitation

Providing answers which are socially desirable remains one of the likely limitation in many scientific studies, and especially so for those which study sensitive topics such as age disparate sex and VAWG. Authors may need to consider this and provide description on whether or not this may have influenced participant’s responses and consequently the results.
