# Supplementary material for: “The child of your fellow is your child”: Building on existing protective norms to engage men as caregivers; qualitative findings from an exploratory evaluation of an edutainment intervention to prevent age-disparate transactional sex
Source: PLoS One. 2025 May 2;20(5):e0321191. doi: 10.1371/journal.pone.0321191 (PMC12048162; doi:10.1371/journal.pone.0321191)
Supplement: Attachment — Submitted filename: Response to Reviewers.docx [file pone.0321191.s005.docx]

25 February, 2025

PLOS ONE – Response to Reviewer Comments

PONE-D-24-32227
“The child of your fellow is your child”: Building on existing protective norms to engage men as caregivers; qualitative findings from an exploratory evaluation of an edutainment intervention to prevent age-disparate transactional sex

| **Comments** | **Author Responses** |
| --- | --- |
| **Editor** | |
| Two referees provided high-quality reports suggesting a range of generally minor revisions.  I will not add to these beyond noting that in referring to the broader literature around engaging men in the prevention of IPV, you could also draw on the recent systematic review I conducted with a team suggesting that engaging men was as effective in reducing and preventing IPV as other strategies (engaging women directly, community mobilization): Leight et al. in the Journal of Global Health, 2024.  Otherwise, the referees have provided useful suggestions to guide your revision. | Thank you for recommending this relevant recent publication, we have added mention to it on pp 4-5, lines 95-97: “Moreover, a recent meta-analysis of 27 randomised controlled trials found that interventions were equally effective at reducing IPV when targeting communities, women only, men only or couples [20], suggesting the need for programming targeting each group.” |
| **Reviewer 1** | |
| This is a very well written paper that makes a strong and unique contribution to the literature. I certainly recommend this paper for publication. The authors clearly justify this paper given the gap in the evidence. I also appreciate the focus on engaging men to prevent adolescent girls' engagement in age disparate transactional relationships. I do have some suggestions to strengthen the paper overall: | Thank you for your careful review and the positive comments, we look forward to addressing your suggestions. |
| The introduction notes that "most research on engaging men in VAWG efforts has focused on men as sexual partners." I would expand this to also reference the wealth of programme and research attention on engaging men as couples in IPV prevention programmes. Indeed, evidence suggests that working with men and women together to prevent IPV can be more effective than working with individual men and women (you could cite for instance Dunkle K, Stern E, Chatterji S, Heise L. Effective prevention of intimate partner violence through couples training: a randomised controlled trial of Indashyikirwa in Rwanda. BMJ Glob Health. 2020 Dec;5(12):e002439. doi: 10.1136/bmjgh-2020-002439. PMID: 33355268; PMCID: PMC7757483) | We have added the proposed sentence and citation on p 4, lines 79-80. |
| Another study to consider citing in the introduction around barriers/limitations of engaging men in fatherhood programmes is a study from Bolivia that did not have an impact on IPV, and a significant reason for this from the qualitative study was the poor engagement of men as fathers: Stern E, Alemann C, Delgado GAF, Vásquez AE. Lessons learned from implementing the parenting Program P in Bolivia to prevent family violence. Eval Program Plann. 2023 Apr;97:102207. doi: 10.1016/j.evalprogplan.2022.102207. Epub 2022 Dec 21. PMID: 3658743 2. Part of the reason for this was not doing good formative research or meeting men where they are; for instance men worked long hours and commuted so had limited time available for the time and location of the parenting programme sessions. I think this can come out too more in the recommendations of the importance of engaging men where they are and trying to address barriers to their engagement/meet their particular needs. | Thank you for sharing this relevant paper. We have added discussion of it in the introduction on p 5, lines 104-105: “It can also be difficult for men who work long hours to travel long distances for programming.”  We have also drawn out the potential solutions you have suggested in the discussion p 31, lines 679-681: “This is in line with previous research on engaging men in VAWG programming, which highlights the importance of providing men with flexible programming that they can engage with at a place and time that is convenient for them [26].” |
| In the introduction, it would also be great if you could describe evidence of additional benefits of engaging men as fathers to prevent both violence against children and IPV? See for example the Bandheberho evaluation in Rwanda: .Doyle K, Levtov RG, Barker G, Bastian GG, Bingenheimer JB, Kazimbaya S, Nzabonimpa A, Pulerwitz J, Sayinzoga F, Sharma V, Shattuck D. Gender-transformative Bandebereho couples' intervention to promote male engagement in reproductive and maternal health and violence prevention in Rwanda: Findings from a randomized controlled trial. PLoS One. 2018 Apr 4;13(4):e0192756. doi: 10.1371/journal.pone.0192756. PMID: 29617375; PMCID: PMC5884496. | We have added this on p 4, lines 90-92: “Additionally, research has found that engaging men as fathers can reduce physical and sexual violence against their partners, and physical punishment of children [17], including in Tanzania [18].” |
| A recent evidence review published by the Prevention Collaborative (2025) also indicates the value of engaging men as fathers to prevent child maltreatment and IPV, as well as other benefits, including to strengthen men's mental health, and strengthen quality of relationships with their children. I think emphasizing this is important to not only consider men as instrumentalist to the benefits of women and children (absolutely important) but also consider the benefits to men through their engagement in such programming. | We have added this on p 4, lines 92-94: “Parenting programmes have also been linked to improved parental mental health, which in turn contributes to reductions in violence and maltreatment of children and strengthened parent-child relationships [19].” |
| In the introduction the authors suggest that joint, or family-wide interventions might be more effective" clarify "than engaging fathers alone"; you could also link this to the benefits of accountability to women and girls through these family wide interventions. | Thanks, we have added this on p 5, lines 117-118. |
| On page 7, I would say 'most programmes have focused on early child outcomes' including to prevent violence against children, as many parenting programmes have violence against children as a primary outcome, and this links it back more broadly to focus on gender transformative programming and age-disparate sexual relationships. | We have added this on pp 6-7, lines 142-146. |
| Existing interventions targeting men as fathers of adolescent girls that I am more familiar with are also ones preventing VAC including preventing child marriage. For instance, there is an ongoing adaptation of the Indashyikirwa programme in Syria which has targeted couples who live with adolescent girls to prevent both IPV and child marriage of the parents' adolescent girls. | Thanks, the ‘Parenting for Lifelong Health’ programme in Tanzania (which you reference below) also targeted men as fathers of adolescent girls. We have added a paragraph describing this intervention in the introduction as an example. |
| The method are very rigorous methods and I commend the very rich findings. | Thank you for these positive comments. |
| In the discussion, I am not sure it's fair to say while men's engagement with parenting programmes is often low, as we have many examples (including the Bandeberho from Rwanda), or this recent scale up of a parenting programme in Tanzania, of successfully engaging men in parenting programmes. I do think it is important to reference the latter study, especially since it is of parenting programmes with adolescent girls: Jamie Lachman, Joyce Wamoyi, Mackenzie Martin, Qing Han, Francisco Antonio Calderón Alfaro, Samwel Mgunga, Esther Nydetabura, Nyasha Manjengenja, Mwita Wambura, Yulia Shenderovich - Reducing family and school-based violence at scale: a large-scale pre–post study of a parenting programme delivered to families with adolescent girls in Tanzania: BMJ Global Health 2024;9:e015472. | Thank you, we have added mention of the Tanzania study in the introduction on p 6, lines 129-140: “There have also been examples of successful parenting interventions in Tanzania. Recently, the ‘Parenting for Lifelong Health programme, which was adapted from the South African context, was found to be effective in reducing child maltreatment, IPV, and parent and child depression, as well as improving sexual health communication between parents and daughters, among other outcomes [23]. The programme was developed to address the underlying drivers of HIV among adolescent girls through a 14-session curricula which was delivered to over 75,000 parents and adolescent girls [23]. Over one-third of the parents included were men, suggesting there is a high demand for parenting programmes from male caregivers in Tanzania. To further scale-up this programme while reducing costs, the researchers developed a phone application to deliver the material to parents on mobile phones, which were distributed throughout communities in the Mwanza region [29]. This ‘ParentApp’ programme is currently being evaluated through a cluster-randomised controlled trial, which is expected to come to completion in 2025 [29].”  We have also added it as a reference in the discussion, and changed the wording from engagement is “often low” to “can at times be limited”. |
| I think you can definitely note many barriers to engaging men as fathers but also lots of examples of programmes that have done this. I would definitely cite Equimundo's recent report on what worked with Programming P to engage men as fathers here for examples of what has been learned about what works to engage men as fathers in parenting programmes. Alemann, Clara, Rachel Mehaffey, and Kate Doyle. 2023. Core Elements of Gender-Transformative Fatherhood Programs to Promote Care Equality and Prevent Violence: A Practitioner Guide. Washington, DC: Equimundo. [equimundo.org/resources/core-elements-of-gender-transformative-fatherhood-programs-to-promote-care-equality-and-prevent-violence/](http://url.uk.m.mimecastprotect.com/s/QoI_CX5WOIxJKxDcksJTWAj07?domain=equimundo.org). | We have added this on p 31, lines 681-687: “Other strategies that have been identified as key to promoting men’s engagement include strong partnerships with implementing partners and in-country stakeholders, creating contextually appropriate content based on formative research, taking the time to adequately train facilitators, engaging influential community members to support normative change and securing funding from flexible donors [59]. These strategies were also adopted by LINEA and are further described in an intervention development paper by Howard-Merrill et al. [43]. |
| In limitations: could you add something about positionality of authors/researchers to the participants and how this was navigated? | We have added this on pp 34-35, lines 769-773: “ This may be, in part, be due to participants long-standing relationship with TCRS, which fostered trust in both the organisation and the study. While the AGO researchers were more educated and came from a more urban area then the participants, being introduced by TCRS and having plenty of experience working with the community likely helped establish credibility and comfort.” |
| The authors' important point around accountability to women and girls is great; perhaps you can also note something around there can be limitations/risks with notion of men protecting girls including controlling behaviours or limiting agency on behalf of girls around who they are interested/in choose to date. It would be great if this is reflected on a bit more in discussion, including perhaps noting in the limitations that you do not have the perspective from adolescent girls themselves. | Thank you for raising these important points. We have added a discussion of these risks on p 34, lines 751-754: “ In supporting this change, practitioners must be careful not to inadvertently promote men’s controlling behaviours by ensuring they are respectful of the agency of adolescent girls’, including, for example those who do choose to date.”  We have also added the limitation on p34, lines 762-765: “While data from adolescent girls were included in our companion papers (see [44, 45]), findings did not speak directly to their perspectives of the role of male caregivers in preventing age-disparate transactional sex, and therefore data from girls were not included in this study.” |
| **Reviewer 2** | |
| I have done the review and the paper is well presented, and a significant contribution to knowledge on the subject. Please see the attached minor comments for review. | Thank you for these positive comments. We look forward to addressing your comments. |
| Authors should consider starting a new paragraph with a strong and direct statement. Therefore, the sentence in Line 63 "Others have also questioned..." should be revised. This comment is also relevant for Line 94 and 115. | In response to this comment we have updated the beginning sentences of these paragraphs. “Others have also questioned…” has been combined with the paragraph above, so the second paragraph begins: “Evaluations of interventions aimed at engaging men…” on p 3 line 61.  We have made similar edits to the beginnings of the following paragraphs on p 5, line 98 and 119. |
| Concept of Transactional Sex: Authors may consider starting the section "Age-disparate transaction sex" Line 131, with basic concepts of the subject presented in later paragraphs - Line (140 -146) and part of the subsequent paragraph (Line 147 -151) before detailing its consequences (HIV and VAW) | We have chosen to keep the current ordering of these paragraphs as we think the paragraph on HIV provides a nice segway from the previous section to discussing age-disparate transactional sex. |
| Line 162 - It notes that the evaluation was carried out in two regions of Tanzania - Kigoma and Shinyanga. I have not seen in the paper the rationale to exclude Kigoma results or focusing in Shinyanga only | We have added a sentence on p 9, lines 201-204 describing why we present the findings from Shinyanga and Kigoma separately, and where the findings from Kigoma have been published. It reads: “Findings from the study conducted in Kigoma region, where the radio drama was broadcast on a local radio station rather than given to participants on a flash drive, are presented in another paper by Howard-Merrill et al. [54].” |
| Contextualization - I lack context specific information of the problem in Tanzania and Shinyanga specifically in regard to age-disparate sex, VAWG and men's involvement as agent for change and care givers. I assume the context may have been described elsewhere as the current paper is indicated to be part of three. However, readers of the current paper will benefit from a brief description of the same | In response to this comment we have added a brief description of Shinyanga region on p 8, lines 180-194: “We selected Kishapu district in the Shinyanga region as the study site for several reasons, including ease of implementation and the prevalence of issues associated with age-disparate transactional sex. The choice was also influenced by a pre-existing relationship with the implementing partner organisation, the Tanganyika Christian Refugee Service (TCRS), which is based in the area. TCRS programming focuses primarily on building community resilience for climate change adaptation and mitigation, and preventing gender-based violence. Shinyanga is a predominantly rural region where agriculture (crop cultivation and livestock rearing) is the main occupation [46]. It has higher than average rates of HIV [47] compared to the national average (4.4%) [48], and among the highest prevalence of IPV in the country [49], with estimates as high as 78% of ever-married women reporting experiencing violence in their lifetime [50]. The region also has the highest prevalence of child marriage in Tanzania, with estimates between 59-64% [50, 51], and a recent study highlighting a culture of tolerance towards sexual violence among married adolescent girls [52]. However, research with caregivers and daughters has also shown strong support for parent-child connectedness as a strategy to prevent early sexual debut among adolescent girls [53].” |
| Methodology  The radio drama "Msichana wa Kati" that was designed to be played in full for nine months (Line 194 - 196) - when did it start? Did it run for nine months as required? On the other hand, authors indicate that baseline evaluation (conducted before the intervention) and endline were 2 -3 months apart (Line 183 -184). There is a date mention in Line 254 that seem to inform the intervention did run long before the baseline assessment - which seem confusing. There is a need for authors to provide clear description on this to warrant intervention replicability. | Thank you for highlighting these important points. As described on p 12, lines 251-256, for this evaluation the participants were provided with a USB flash drive of the radio drama and visited by facilitators to conduct household discussion sessions once per week. In response to this comment we have clarified on lines 252 that participants were also instructed to listen to one episode per week.  We have also clarified that participants were given the flash drive in September 2021. On line 254 it states that participants were recruited 21-27 July 2021, however, due to delays baseline data collection did not occur until September, and the flash drives were distributed after that. This has been clarified on p 14, lines 283-284. |
| Discussions  Structured household discussions and its impact - While the authors have indicated (in response to earlier review comment comment 2) that discussions had no added impact, the team presents conflicting conclusions in line 655 - 659 and recommendation Line 659 - 661. | Thank you for highlighting this inconsistency. While this study does not provide sufficient evidence to support the added value of structured household discussion sessions, it is important to note that this was a small qualitative study. Based on existing literature, we believe that these discussions may play a supportive role in facilitating engagement, and encouraging conversations that might not have occurred otherwise. However this effect might differ depending on context and we acknowledge that more research is needed. To clarify this point, we have revised the discussion on pp 31-32, lines 688-689:  Although this small qualitative study did not find direct evidence of additional impact from structured household discussion sessions, they may have helped facilitate conversations that might not have otherwise occurred, and provided caregivers with a model for initiating and engaging in such discussions. Communication theory suggests that discussions about programming serve as an important, indirect pathway to behaviour change as they indicate that others within one’s social group are engaging with the intervention messages [60]. However, a recent review of edutainment programmes aimed at preventing violence against women and children found mixed evidence on whether discussion sessions contribute additional impact beyond the edutainment intervention itself [61]. Some researchers have hypothesized that this may be because discussions occur organically within communities, particularly when interventions are delivered in group settings, reducing the need for structured sessions facilitated by implementers [62]. Given these mixed findings, further research is needed to assess the true impact of discussion sessions, particularly in comparing individual and group-based delivery modalities. |
| Limitation  Providing answers which are socially desirable remains one of the likely limitation in many scientific studies, and especially so for those which study sensitive topics such as age disparate sex and VAWG. Authors may need to consider this and provide description on whether or not this may have influenced participant’s responses and consequently the results. | We agree with the reviewers point, and have addressed this on pp 34-35, lines 766-776 with the following addition: “There is always a possibility that participants provide socially desirable responses, particularly when discussing sensitive topics such as age-disparate transactional sex. We acknowledge this potential bias, although we found no direct evidence of it. This may be, in part, be due to participants long-standing relationship with TCRS, which fostered trust in both the organisation and the study. While the AGO researchers were more educated and came from a more urban area then the participants, being introduced by TCRS and having plenty of experience working with the community likely helped establish credibility and comfort. Although it is possible that participants underreported their engagement in age-disparate transactional sex, this would not have affected the primary findings of this study, which focus on broader changes in male caregivers following the intervention. However, we recognise that social desirability bias may have led some participants to overstate the programmes effects.” |

REFERENCES

17.	Doyle K, Levtov RG, Barker G, Bastian GG, Bingenheimer JB, Kazimbaya S, et al. Gender-transformative Bandebereho couples’ intervention to promote male engagement in reproductive and maternal health and violence prevention in Rwanda: Findings from a randomized controlled trial. PLOS ONE. 2018;13(4):e0192756. doi: 10.1371/journal.pone.0192756. PubMed PMID: 29617375.

18.	Lachman J, Wamoyi J, Martin M, Han Q, Alfaro FAC, Mgunga S, et al. Reducing family and school-based violence at scale: a large-scale pre–post study of a parenting programme delivered to families with adolescent girls in Tanzania. BMJ Glob. Health. 2024;9(11):e015472. doi: 10.1136/bmjgh-2024-015472. PubMed PMID: 39581633.

19.	Prevention Collaborative. Improving the mental health of caregivers as a strategy to prevent family violence: What does the evidence suggest? Amherst, MA: Prevention Collaborative; 2025.

20.	Leight J, Cullen C, Ranganathan M, Yakubovich A. Effectiveness of community mobilisation and group-based interventions for preventing intimate partner violence against women in low-and middle-income countries: a systematic review and meta-analysis. JoGH. 2023;13. doi: 10.7189/jogh.13.04115. PubMed PMID: 37861113.

23.	Siu GE, Wight D, Seeley J, Namutebi C, Sekiwunga R, Zalwango F, et al. Men’s involvement in a parenting programme to reduce child maltreatment and gender-based violence: Formative evaluation in Uganda. EJDR. 2017;29(5):1017-37. doi: 10.1057/s41287-017-0103-6.

26.	Stern E, Alemann C, Delgado GAF, Vásquez AE. Lessons learned from implementing the parenting Program P in Bolivia to prevent family violence. Eval Program Plann. 2023;97:102207. doi: 10.1016/j.evalprogplan.2022.102207. PubMed PMID: 36587432.

29.	Baerecke L, Ornellas A, Wamoyi J, Wambura M, Klapwijk J, Chetty AN, et al. A hybrid digital parenting programme to prevent abuse of adolescents in Tanzania: study protocol for a pragmatic cluster-randomised controlled trial. Trials. 2024;25(1):119. doi: 10.1186/s13063-023-07893-x. PubMed PMID: 38351094.

43.	Howard-Merrill L, Zimmerman C, Sono R, Riber J, Wamoyi J, Pawlak P, et al. Shifting social norms to prevent age-disparate transactional sex in Tanzania: what we can learn from intervention development research. Front. Psychol. 2023;14:926531. doi: 10.3389/fpsyg.2023.926531. PubMed PMID: 37205068.

44.	Pichon M, Carter DJ, Howard-Merrill L, Sono R, Gimunta V, Rutenge O, et al. A mixed-methods, exploratory, quasi-experimental evaluation of a radio drama intervention to prevent age-disparate transactional sex in Tanzania. Front. Reprod. Health. 2022;4:1000853. doi: 10.3389/frph.2022.1000853. PubMed PMID: 36531443.

45.	Pichon M, Buller AM, Gimunta V, Rutenge O, Thiaw Y, Sono R, et al. Qualitative evaluation of an edutainment intervention to prevent age-disparate transactional sex in Tanzania: Changes in educational aspirations and gender equitable attitudes towards work. PLOS Glob. Public Health. 2024;4(4):e0002527. doi: 10.1371/journal.pgph.0002527. PubMed PMID: 38568918.

46.	The United Republic of Tanzania (URT), Ministry of Finance, Tanzania National Bureau of Statistics and President’s Office - Finance and Planning, Office of the Chief Government Statistician, Zanzibar. The 2022 Population and Housing Census: Shinyanga Region Basic Demographic and Socio-Economic Profile Report. Tanzania; 2024.

47.	UNAIDS. Country progress report - United Republic of Tanzania: Global AIDS Monitoring 2020. Geneva, Switzerland; 2020.

48.	The United Republic of Tanzania, Prime Minister's Office, Tanzania Commission for AIDS. National HIV Prevention Road Map 2023/24-2026/27. Dar es Salaam, Tanzania; 2024.

49.	Nyato D, Materu J, Kuringe E, Zoungrana J, Mjungu D, Lemwayi R, et al. Prevalence and correlates of partner violence among adolescent girls and young women: Evidence from baseline data of a cluster randomised trial in Tanzania. PLoS One. 2019;14(10):e0222950. doi: 10.1371/journal.pone.0222950. PubMed PMID: 31593577.

50.	The World Bank. Tanzania gender based violence assessment: Scope, programming, gaps and entry points. Washington, DC: The World Bank; 2017.

51.	MoHCDGEC (Ministry of Health, Community, Development, Gender, Elderly and Children). Child marriage in Tanzania at glance. Dar es Salaam: Ministry of Health, Community Development, Gender, Elderly and Children; Tanzania; 2017.

52.	Mwanukuzi C, Nyamhanga T. "It is painful and unpleasant": experiences of sexual violence among married adolescent girls in Shinyanga, Tanzania. Reprod Health. 2021;18(1):1. doi: 10.1186/s12978-020-01058-8. PubMed PMID: 33388066.

53.	Shigela DN. Parents’ role in fighting premarital sexual intimacy in Tanzania: A case of secondary schools in Shinyanga rural district. The Open University of Tanzania. Dar es Salaam, Tanzania; 2018.

54.	Howard-Merrill L, Pichon M, Witt A, Sono R, Gimunta V, Hofer E, et al. "I have learned that nothing is given for free": an exploratory qualitative evaluation of a social norms edutainment intervention broadcast on local radio to prevent age-disparate transactional sex in Kigoma, Tanzania. BMC Public Health. 2024;24(1):2907. doi: 10.1186/s12889-024-20440-w. PubMed PMID: 39434074.

59.	Alemann C, Mehaffey R, Doyle K. Core elements of gender-transformative fatherhood programs to promote care equality and prevent violence: Results from a comparative study of Program P adaptations in diverse settings around the world. Washington DC: Equimundo; 2023.

60.	Arias E. How does media influence social norms? Experimental evidence on the role of common knowledge. Political Sci Res Methods. 2019;7(3):561-78. doi: 10.1017/psrm.2018.1.

61.	Peterman A. Edutainment to prevent violence against women and children. SSRN. 2024.

62.	Hidrobo M, Dione M, Heckert J, Le Port A, Peterman A, Seye M. C’est la vie!: Mixed impacts of an edutainment television series in West Africa. SSRN. 2025. doi: 10.2139/ssrn.5097718.
